# Can payment by results ensure equitable access to contraceptive services? a qualitative case study

**DOI:** 10.1186/s12939-023-01919-1

**Published:** 2023-05-28

**Authors:** Victoria Boydell, Joseph Holden, Ginny Robins, Joyce Mumah, Barnabas Abok, Sandra Mudhune, Caroline Guinard, Heidi Quinn, Meghan Bishop

**Affiliations:** 1 University of Essex, Wivenhoe Park, Colchester, CO4 3SQ UK; 2 Foresight Development Associates, The Greenhouse N16, 49 Green Lanes, London, N16 9BU UK; 3 MSI Reproductive Choices, 1 Conway Street, Fitzroy Square, London, W1T 6LP UK; 4 International Planned Parenthood Federation, CVS Plaza, 5Th Floor, Kasuku Road, Off Lenana Road, P.O. Box 30234, Nairobi, 00100 Kenya; 5 Oxford Policy Management, Clarendon House, Cornmarket Street, Oxford, OH1 3HJ UK

**Keywords:** Payment-by-results, Performance-based financing, Sexual and reproductive health, Equity

## Abstract

**Background:**

The Leave No One Behind (LNOB) agenda compels sexual and reproductive health and rights (SRHR) implementers to focus on the multiple and intersecting forms of discrimination and inequalities. One strategy to address these is Payment by Results (PbR). Using the Women’s Integrated Sexual Health (WISH) programme as a case study, this paper examines if and how PbR can ensure equitable reach and impact.

**Methods:**

Given the complexity of PbR mechanisms, a theory-based approach was used in the design and analysis of this evaluation, drawing on four case studies. These were conducted by reviewing global and national programme data and by interviewing 50 WISH partner staff at national level and WISH programme staff at global and regional levels.

**Results:**

The case studies found that inclusion of equity-based indicators in the PbR mechanism had demonstrable effects on people’s incentives, on how systems work, and on modes of working. The WISH programme was successful in achieving its desired programme indicators. The use of Key Performance Indicators (KPIs) clearly incentivised several strategies for service providers to innovate and reach adolescents and people living in poverty. However, there were trade-offs between performance indicators that increased coverage and others that increased equitable access, as well as several systemic challenges that limited the possible incentive effects.

**Conclusions:**

The use of PbR KPIs incentivised several strategies to reach adolescents and people living in poverty. However, the use of global indicators was too simplistic, resulting in several methodological issues.

## Background

In 2015, governments who signed on to the 2030 Agenda for Sustainable Development pledged to “leave no one behind” (LNOB) by eradicating poverty, ending discrimination and reducing inequalities [1]. The LNOB agenda compels sexual and reproductive health and rights (SRHR) implementers to focus on the multiple and intersecting forms of discrimination and inequalities that people face. One strategy to direct implementers in these efforts is performance-based financing (PBF): that is, the use of mechanisms to link funds or payments to the achievement of specified results (such as reaching excluded populations).

Programmes where payments are made after the achievement of pre-agreed outputs and outcomes, often covered by the term Payment by Results (PbR), have been introduced widely in recent years to help achieve specific outcomes in health services [2]. Purchasing mechanisms that use performance incentives are increasingly being adopted to seek to improve the quantity and quality of healthcare services. Several quasi-experimental studies examining the effects of PbR on contraceptive services found positive outcomes in Congo, Rwanda, Zambia, and Burundi [3–8], whilst other studies have found no or limited effects [9, 10]. However, equity effects in service provision have yet to receive significant consideration [11, 12] despite some attempts to combine PbR with equity measures that target excluded and vulnerable groups [11, 13–15]. This is especially true in specific health sectors: for example, a recent systematic review of financing for family planning (FP) highlighted an evidence gap around the effects of PbR on equitable access and FP-related outcomes [16]. For this reason, this article analyses how and to what extent the inclusion of equity-based indicators in the PbR mechanism used for the Women’s Integrated Sexual Health (WISH) programme improved its equitable reach and impact.

The WISH programme (2018–2021 and extended to March 2024), funded by the United Kingdom’s Foreign, Commonwealth and Development Office (FCDO^1^) supported women’s and girls’ full, free and informed choice to use family planning/modern methods of contraception. The programme’s key performance indicators (KPIs) included equity-related indicators to measure reaching people living in extreme poverty and adolescents. These equity-related indicators sat alongside other KPIs of additional users of FP (AUs) and couple years of protection (CYPs), as well as sustainability. All these KPIs were integrated into the programme’s PbR mechanism. The equity KPIs were intended to motivate partners to increase the service coverage to adolescents and those living in poverty, and to incentivise programming to actively reach them.

The WISH programme was structured under two lots: Lot 1, contracted to a consortium led by MSI Reproductive Choices, operating in 12 countries in West and Central Africa; and Lot 2, contracted to a consortium led by the International Planned Parenthood Federation (IPPF), operating in 15 countries in Eastern and Southern Africa and three countries in South Asia.^2^ The programme was supported by a third-party monitoring body, WISH4Results that assessed the quality of provision of care, including contraceptive counselling. A PbR mechanism links the partners’ fees to performance against six KPIs defined at the start of the programme, shown in Table 1 below.

**Table 1:** Definition of the Key Performance indicators at the start of the programme^a^

Key performance indicator (KPI)	Definition of the KPI
Couple Years of Protection (CYP)	Number of CYPs generated by family planning (FP) services (across each country programme, no single country to provide > 40% CYPs
Additional Users (AUs)	Number of AU’s reached by end of project (across the portfolio)
Poverty^b^	Number of FP service users living on less than USD 1.90 a day which is in parity with the national average % poverty headcount per country by end Year 2
Youth (a) (5% country target)^b^	Minimum of 5% of FP clients are under 20 per country per annum
Youth (b) (15% portfolio target)^b^	Minimum average of 15% of FP users under 20 across the whole portfolio by December 2020
Sustainability	At least two sustainability milestones achieved in at least 75% of eligible countries per annum

^a^WISH use the term youth when describing the KPI and this refers to adolescent populations, therefore we use the term *youth* when we are referring to the KPI

^b^Equity indicators

Source: Information directly provided by implementing partners

The first two PbR KPIs relate to CYPs and AUs, both quantitative measures that relate to coverage. Approximately 60 percent of the PbR value was linked to these two indicators. The sustainability PbR KPI aimed to improve the supply of integrated sexual and reproductive health (SRH)/FP services, to sustain demand for these services, and/or to catalyse improvements to the enabling environment for integrated SRH services. The design of WISH included three equity PbR KPIs that explicitly aimed to promote a leave no-one behind agenda. The PbR KPIs were also included within the programme’s logical framework (log frame) indicators, either as outcomes or outputs, and were specified alongside other important (non-PbR) goals of the programme including, for example, quality of care, and on comprehensive choice of FP methods. In addition, WISH strongly emphasised the inclusion of people with disabilities (PWD) in its design and through the inclusion of an (non-PbR) output indicator in the log frame.

Using the WISH programme as a case study, this paper examines if and how PbR can ensure equitable access to contraceptive care. A programme theory of how PbR equity mechanisms may function within WISH is used as a framework to assess four case studies from the WISH programme. The findings are then situated in the wider literature on PbR and equity.

## Methods

**Fig. 1:**
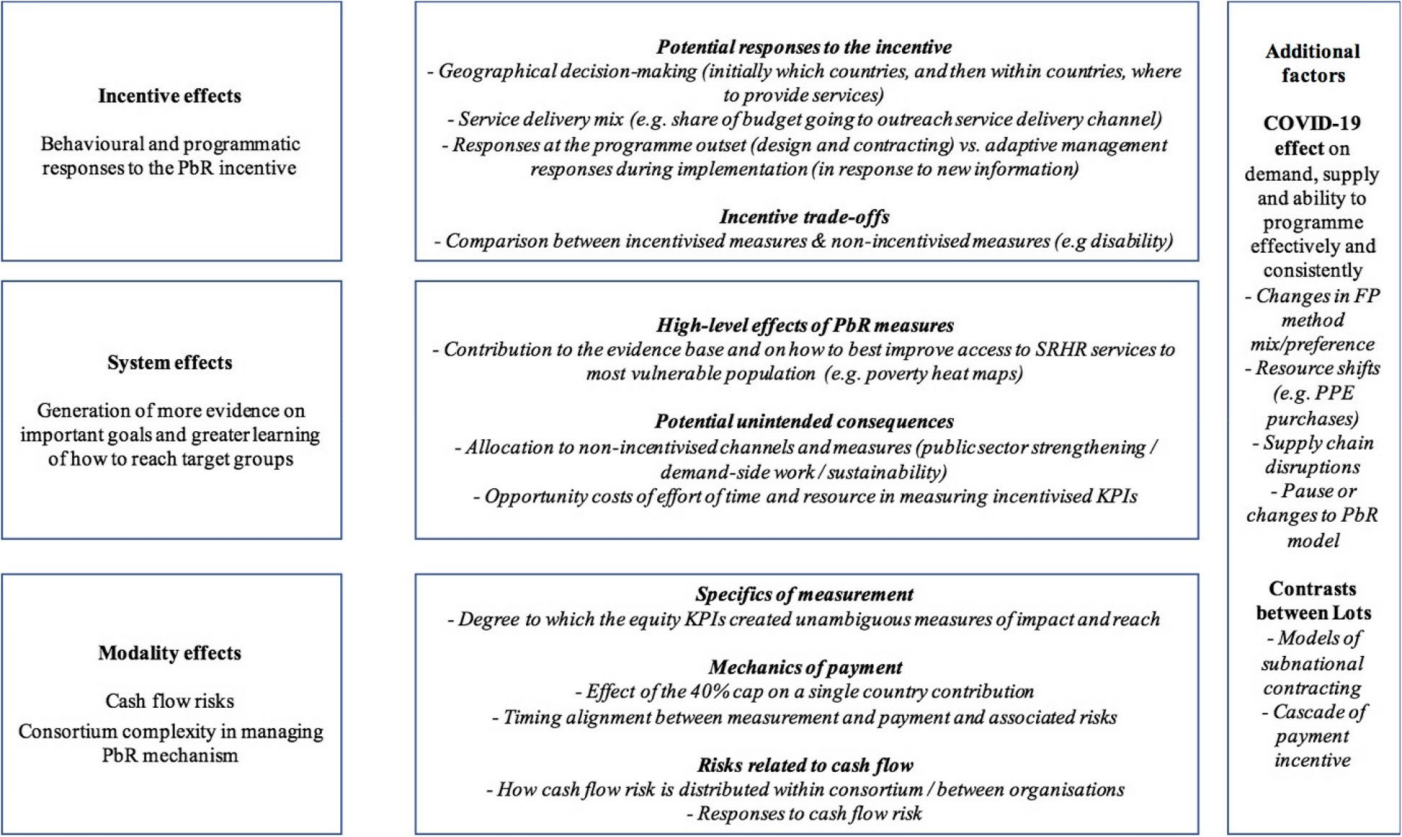
Research framework of how PBR equity mechanisms may function within WISH

Given the complexity of PbR mechanisms, a theory-based approach was used in the design of the study and drew on four case studies composed of several data sources (Craig et al 2008) [17]. The analysis used a standard theoretical modelling of PbR as a solution to the principal-agent problem (see for example Grittner, 2013) [18], with an entity carrying out an action (the agent) on behalf of an entity paying for the action (the principal). The ‘problem’ is that the agent may have different goals from the principal with respect to the action or service that they are being paid to undertake – they may therefore not put in the effort required to achieve the goal, or instead pursue their own different goals. A financial incentive is introduced to address this problem and aims to align goals and incentives for the agent to act accordingly. This incentive aims to rectify two types of potential imbalance between the principal (in this case, FCDO) and the agent (in this case, WISH implementing partners): (1) an imbalance in priorities (which goals the agent should pursue), and (2) an imbalance in information (on which actions are pursued and their relative cost). The principal-agent model provides a structure to describe PbR and how it seeks to incentivise the achievement of results. Figure 1 sets out the research framework for how PbR equity mechanisms may function within WISH in terms of the potential effects of PbR.

Based on this framework, this study examined three of the PbR mechanisms:

1. **Incentive effects**: the behavioural and programmatic responses to the PbR incentives – these are the main means by which WISH partners may have responded to the KPI PbR incentive in order to better achieve equity.
2. **System effects**: including broader potential benefits and risks of including the PbR measures. These can relate to the evidence generated via PbR KPIs and any transformative potential of these – for example, whether equity was incorporated into learning and whether evidence generated improved understanding of ‘what works’ to reach particular communities.
3. **Modality effects**: including elements of the PbR contracting modality as opposed to other modalities. These include the challenges and risks related to measurement and to cash flow from the PbR mechanism, and how the partners responded to these.

The framework also sets out important ‘additional factors’ under consideration, including the adaptations required by the COVID-19 pandemic, and the potential for contrasts between the two WISH lots, in terms of the models of operation (e.g., degree of top-down control and degree to which the payment incentive(s) cascade to subnational partners).

A global case study (covering the experience of both WISH lots) and three country case studies (Senegal, Ethiopia and Pakistan) captured a range of performance against the equity-related KPIs. The country case studies featured one country from each of the WISH lots and one country in which both partners were working. Case studies were conducted by reviewing global and national programme monitoring data and organising semi-structured key informant interviews (KIIs) with WISH partners at national level and WISH programme staff at global and regional level.

Data were collected from March to October 2021. Three national consultants were trained by the lead consultant in the study. Due to the ongoing COVID-19 pandemic, interviews were arranged remotely via telephone, Zoom or Skype, as was convenient for the respondents. Interviews were conducted in English or the appropriate national language and translated afterwards where necessary by the national consultants. A total of 50 individuals were interviewed (see Table 2).

**Table 2:** Respondent type and number, by case study

Case study level	International or regional office	Country headquarters	Service delivery	Total
Global case study	FCDO (5), MSI (5), IPPF (4); TPM/W4R (5), Partners (2)	-	-	**21**
Senegal case study	-	5	4	**9**
Ethiopia case study	-	7	3	**10**
Pakistan case study	-	5	5	**10**
Total	**21**	**17**	**12**	**50**

Because the evaluation was undertaken as a monitoring, evaluation and learning activity and adheres to the *AEA guiding principles for evaluators (2004),* ethical approval was not sought for this study. All organizations were engaged in reviewing the protocol, data collection instruments and consent forms to ensure they were appropriate and posed minimal risks for everyone involved. Ethical principles and standards were adhered to in conducting the evaluation, including obtaining informed consent from participants, maintaining participants’ privacy and confidentiality, complying with data protection legislation, and identifying potential risks to participants as well as mitigation strategies. All respondents received an informed consent form that included information about the purpose of the study and assured confidentiality. Each participant provided verbal consent prior to the interview and in some cases permission to audio-record. Respondents participated freely and were allowed to withdraw their participation at any point during the interview and study.

For each case study, the data from the document review and KIIs was synthesised around the elements of the research framework detailing the three main types of effects: incentive effects, systems effects, and modality effects. The findings for each element were then compared across the case studies to identify cross-cutting findings.

## Results

The findings from the study are set out against the three PbR mechanisms outlined in the research framework: incentive effects, systems effects, and modality effects.

### Incentive effects

During the design stage of WISH, Department for International Development (DFID) advisors felt that the ‘quantity’ focus of coverage targets required offsetting to ensure that disadvantaged populations were reached and that other goals, such as sustainability, be included. DFID^3^ viewed its main remit to be poverty reduction,^4^ and therefore held that WISH should reach the poorest in each country. It was also clear that young people (defined as those under 20 years of age) face particular barriers to accessing SRH/FP services, especially the potential of stigma. PbR was therefore explicitly linked to the goal of improving reach to the poorest and to adolescents.

The tender document set out that PbR would be applicable and provided a list of countries where the programme could operate, inviting applicants to specify where they would operate.^5^ The successful consortia came in with the full list of countries in the tender, and with a maximum proportion of fees (100%) linked to the PbR KPIs. The scoring structure in the Invitation to Tender (ITT) incentivised the applicants to include the maximum number of countries possible and to place a large proportion of fees with the PbR mechanism and hence at financial risk.

For both lots, consortia partners used their own historic data to model what could be confidently expected from the programme in terms of delivery against the PbR KPIs, where that data was available. There was not necessarily up-to-date national data on the poverty status of many of the countries in both lots. For Lot 2 (led by IPPF), the lack of baseline data on FP service users’ poverty status was particularly challenging. Both consortia felt that it was not clear how reliable, comparable, and representative the poverty KPI would be. Another issue was trying to identify a single indicator, particularly for adolescents, that could be relevant to different demographic contexts: for example, a very ambitious target in the South Asia context would likely be unambitious in Sahelian countries (due to demographics).

The WISH programme was hit by the COVID-19 pandemic 17 months into implementation. The national lockdowns, closures of facilities, commodity procurement and supply challenges, as well as the need to provide services safely with new social distancing procedures, led to two adaptations: (1) a COVID waiver in 2020, and (2) a ‘post-COVID’ waiver in 2021. The former led to the suspension of PbR for six months, while the latter gave partners the opportunity to make a case for waiving some PbR risk based on how COVID-19 made the achievement of the PbR KPIs less feasible. In both cases, waivers had the greatest effect in addressing risk relating to the poverty PbR KPI (i.e., eliminating the financial risks from the relative underperformance on this KPI).

Even in the COVID-19 context, the use of PbR KPIs clearly incentivised the programme to make a significant effort to reach adolescents and people living in poverty. Several strategies had been put in place to reach these specific communities in the three case study countries. To reach more adolescents, strategies included training of service delivery staff and mobilisation activities aiming to locate services closer and more conveniently for the adolescent clientele, while also providing services that were appropriate to younger people (e.g., extending opening hours to offer services on evenings and weekends). Other strategies included direct outreach in universities and industrial parks, adolescent group sessions, engagement of boys and men at community level and through mass media, peer-to-peer approaches, and the use of an outreach service delivery channel to proactively reach young people.

The COVID-19 context makes it difficult to estimate the effectiveness of these strategies on achieving KPIs. For the Youth PbR KPIs, the country and portfolio goals were met. However, there was no increasing trajectory of adolescent access over the three years of the programme – i.e., the average proportion of FP users under 20 years of age neither increased nor decreased significantly for either lot. The first Youth PbR KPI of 5% of service users to be under 20 years of age applicable for all countries was easily met for most countries, though some countries made considerable efforts to reach this indicator. The second Youth PbR KPI of 15% across the consortium was also reached by both lots. This was more ambitious and several countries remained under this threshold.^6^ However, both Youth KPIs illustrate the limitations of using a single indicator across countries with very different contexts. There are reasons to suggest programming for young people became more difficult during COVID-19 lockdowns in several contexts, not least with the widespread school and university closures across countries, through which some awareness-raising interventions were focused.

Strategies adopted by WISH to reach more people in poverty included programming decisions about where to geographically locate services, the removal of service fees, non-discrimination training and a no-refusal policy. At the global level, most stakeholders mentioned the use of poverty-mapping heat maps for locating services and, in a few instances, these heat maps were used to close sites in areas that did not serve excluded populations and open sites in entirely new geographies.

Despite developing strategies in response to the PbR mechanisms, many countries did not meet their poverty PbR KPI in either the first or second year. The KPI was defined according to national benchmark rates, as per the World Bank’s absolute poverty threshold of USD 1.90 Purchasing Power Parity (PPP) per day.^7^ Each country would measure service users who should be in poverty in proportion to the national benchmark level to achieve the PbR KPI.^8^ The poverty PbR KPI had the most significant performance shortfall across the PbR KPIs and carried significant financial risk. This underperformance was in part attributed to the measures used. The most common measure used was the Poverty Probability Index (PPI), which estimates the poverty propensity of a given population using a small number of questions for a survey sample. The PPI was originally designed as an ‘ease of use’ measure for a household survey setting because of its features: it is a quick to collect, easy to calculate and low cost to administer, rather than a precise and accurate, measure (Schreiner, 2018) [19].

The PPI and Multidimensional Poverty Index (MPI) are well-established estimates of poverty. However, they both use their unique metrics and baselines and therefore come to different results when measuring the same sample. They also use benchmark national surveys that are often out-of-date (as early as 2009) and do not reflect the contemporary situation. In addition, by using national benchmarks that covered all ages, the poverty rates of women of reproductive age were likely to be overestimated, making it more difficult to achieve the KPI.^9^ Administering the MPI in a healthcare exit interview setting rather than a household survey setting brought its own challenges. For example, it was not possible to collect the anthropometric data to compute the nutrition indicator. This was mitigated by not measuring nutrition status and instead doubling the weight of the other health sub-indicator (child mortality).

As these methodological issues became apparent with the PPI, Lot 1 (MSI) shifted from using the PPI measure to capture the poverty data in the first and second year to using the MPI measure in the third year. Other changes were made, for example, only using the national benchmark average as it applied to women of reproductive age and dropping the nutrition measures that required close physical proximity to collect during COVID-19. Despite the efforts to address these methodological issues, there remain ambiguities, and this creates a degree of uncertainty about the actual performance on poverty in the WISH programme.

More broadly, the programme was successful in achieving the quantity-focused KPIs (CYPs and AU), and the sustainability PbR KPI. These quantity targets were associated with the greatest financial values, and many respondents felt these were prioritised whilst implementing WISH. For some respondents, the CYP targets were too high, adding significant pressure to their work. For other respondents, the competing PbR KPIs created a cost-effectiveness trade-off as the more remote geographical areas may have offered greater opportunities for reaching people in poverty and addressing unmet need for contraception but would do less well on the CYP goals at the same cost. However, for some respondents, the strategies for achieving the PbR KPIs were seen to be complementary: the same activities that were promoting adolescents or reach to people living in poverty would also be beneficial for the AU and CYP KPIs. Reaching people with disability was viewed by most stakeholders as a success of the WISH programme; however, because reaching people living with disability was only included as log frame indicator and not as a PbR KPI, this study was not able to assess its effects compared to the equity PbR KPIs - youth and poverty.

### System effects

Across the programme, the PbR mechanism was viewed as being largely positive and respondents were highly motivated by the programme level incentives: *“These kinds of approaches have a motivational quality. That is because once you understand that you need resources to run your programmes, but you [sic organization] only get paid once you do the work, you will have the motivation to do a better job and to strategize different approaches and interventions for your work.”*

Other associated improvements are in the data, learning and evidence systems developed to meet the reporting requirements of the WISH programme. This included the data management systems and the client exit interview (CEI) data. Planning systems were also enhanced: PbR brought a clearer approach to planning to achieve results, including how this led to the development of strategies to address inequity and respond to new information as and when it was available. One respondent stated: *“[PbR] makes you work, in order to bring the required result, you plan different strategies based on available options, and by executing different activities in different ways. So, in order to get the required result, it makes you focus and work with effort by implementing different strategies, because if there is no result there is not going to be any payment.”* The PbR KPIs were not seen as detracting or diverting attention from other priorities such as quality of care, availability and informed choice as this was embedded within existing protocol and procedures, log frames and professional training.

There were several systemic challenges that limited the possible incentive effects of the PbR KPIs. Decisions on where to geographically locate service sites are complicated (and not entirely within the powers of the implementing partners, as this is overseen by local health authorities), and the idea of being able to continue to adapt service locations in response to new information on poverty was neither realistic nor necessarily desirable. In Ethiopia, one respondent stated: *“More than half of the outreach clients are below the poverty line. The public facilities support is static. We can’t move them. We can’t change location once we start providing support.”* In contexts where there are no other providers, there are also ethical implications with respect to (re)moving outreach services. As one respondent stated: *“You give women implants, it’s harder to take one out, you want to deliver that”,* but *“how must those women feel, when those services aren’t there?”.* There were, therefore, both ethical and contextual restrictions driving the selection of sites and restricting the ability to change sites once they were set, limiting adaptive programming.

### Modality effects

A distinguishing feature of PbR as a contractual mechanism is that finance is at risk. For any PbR mechanism to be credible, there must be a genuine risk of non-payment and financial loss to the agent organisation and for suppliers to be rewarded for achieving beyond the required performance. For WISH, the total financial risk (‘fees retained for PbR’) was shared across the consortia, based on financial modelling and negotiations at the start of the programme. The risk allocation within consortium partners was complex and required significant negotiation and diplomacy amongst partners in terms of both understanding the risk involved and risk tolerance, particularly for those without prior experience of PbR.

The final financial outcome for PbR was found to be likely neutral (i.e., there were overall no losses incurred). There was a risk that the programmes would run at a loss, though this did not happen. The potential impacts on cashflow due to underperformance on the poverty PbR KPI results were bridged by timely performance on the other KPI payments, particularly the Youth KPIs. Further, there was a degree of flexibility in the PbR mechanism granted by FCDO due to COVID-19: the COVID-19 waiver in 2020 and the ‘post-COVID’ waiver in 2021, combined with careful cashflow management by the consortia, ensured that fees were not lost across the two lots. The flexibility represented by the waivers ultimately meant that the poverty KPI shortfalls would not lead to fees being withheld.

Financial risk was not standard across the consortia. How risk was cascaded to different institutional levels varied, and consortia members negotiated how risk was pooled, which meant that partners faced different degrees of risks. For example, the Youth (b) PbR KPI – the 15% average of reach to those aged under 20 across the portfolio – was achieved across the Lot 2 consortium early in the programme. However, one partner had chosen not to pool risk with other members at the negotiation phase and uniquely faced financial risk. The different ways that the financial and cashflow risk were distributed, including PbR being flexibly applied across consortium partners, worked to protect the cashflow of the organisations. This assumes the ability to pay for the services upfront; and only large, well-resourced organisations would be able to meet these criteria.

The PbR reporting (and its verification) itself represented a significant time and cost burden for team members involved, and therefore an opportunity cost, though this is difficult to quantify. This included the time and energy spent in PbR modelling and analysis, managing risk within the consortia, as well as in negotiating aspects of the PbR with FCDO*.* While *“making sure we have quality in our data is a priority”*, the processes of verification via the third-party monitoring body also require *“a lot more documents, bureaucracy, *etc*.”* Furthermore, *“this takes a lot of people’s time. Then we have calls, to explain processes, *etc*., and this is a burden for us to explain and provide more information.”* The reporting requirements of the PbR generated stress, one respondent stated*: “[It] is also a stressful experience requiring a lot of effort to implement and collect the evidence because the programme is performance-based to get pay-outs.”*

There were positive consequences on ways of working among the partners and the two consortia, including the rich learning exchange between the two lots, particularly between MSI and IPPF. An effect of this collaboration was in aligning changes and contractual amendments between the two lots over the course of WISH, including the discussions on poverty methodologies. In practice, while the two lots tended to converge around mutually beneficial contract and methodological amendments, differences between the two lots remained due in part to different preferences and organisational modus operandi. It is not clear how much additional time and cost was generated by negotiating with both lots for FCDO itself, although it is likely the amount of time and cost diminished over time because the two lots collaborated and presented a unified approach that may have reduced the overall transaction costs.

## Discussion

The WISH programme clearly demonstrated that it is feasible to use a PbR approach to improve equitable access to contraceptive services. The programme was successful in achieving its desired coverage (CYPs and Additional Users), and the use of PbR KPIs clearly incentivised several strategies to reach adolescents and people living in poverty. The Youth KPIs were realised, yet many countries did not meet the poverty PbR KPI, which carried significant financial risk. Other equity outcomes were not associated with KPIs, particularly for those living with disability, and therefore fell outside the scope of the PbR framework. In these cases, it was not possible to compare the effects on access to contraceptive services against the PbR KPI. Yet, overall, there is evidence that, as per the theory, the PbR aligned some equity goals of the principal and agents under the programme. The findings, though, are inconclusive as to whether this programme led to improvements in equity.

These results align with some findings from the broader literature: in particular, the effects of PbR on equity were varied. Existing literature found PbR to have little effect on extreme poverty (those in the lowest quintiles) [13, 15, 20, 21] but to have positive effects on rural–urban inequalities [14]. Notably, many studies argued that PbR interventions tended to be clinic- or facility-based and did not necessarily address structural health system challenges [14, 22], including: the large out-of-pocket expenditure for the ultra-poor [23], bias interpreting how to apply the criteria [15], and more practical barriers such as lack of transport and lack of awareness of the benefits [20]. Likewise, the strategies in WISH were focused on clinic- or outreach service-based, and this may have limited their effects. Strategies to address inequity in supply and service provision may well improve coverage and quality of care but do not address the demand-side efforts that could better reach the most marginalised populations and help overcome inequalities (e.g., [13, 21, 24]).

In programmes focused on improving equitable access to services, a balance is sought between trying to increase coverage and providing equitable provision. This can create inevitable trade-offs when trying to achieve the right equilibrium between quantity, equity, and sustainability, despite their complementarities, especially regarding the relative costs faced for reaching different groups (particularly due to a quantity and equity trade-off) and the types of actions required to achieve different goals (for example, subsidising services, which may include trade-offs between sustainability and achieving long-term changes to health systems).

In similar studies of the equity effects of payment-based results, there were issues with the measurement used. This was also the case with the WISH programme, which used global indicators (e.g., the PPI and MPI methodologies). These indicators were difficult to apply in different demographic contexts. In addition, the benchmarks for these measures were often outdated. This resulted in different degrees of difficulty for countries in achieving their target, particularly for adolescent indicators, given that a standard proportion was used (e.g., 5% or 15% of users in the adolescents category). The consideration of PbR KPIs should go beyond ‘technical’ discussions around data (e.g., on PPI and MPI metrics and mapping methodologies, discussions about site selection, statistical relevance and representation, programming approaches, etc.) to include the opportunity cost of such discussions and the need for better measures. As others [13] have found, the use of pre-determined equity measures (e.g., PPI) with PbR may not necessarily close the inequity gap as intended because these indicators may not correspond to locally relevant forms of disadvantage: there may be more pressing forms of disadvantage that are not considered. The technical challenges in measurement may also simply make them inappropriate for assessing service delivery programme reach.

The poverty metrics used on WISH (MPI and PPI) may have provided useful information for implementing partners, and most partners cited high degrees of learning. However, the combined use of a poverty PbR KPI and of national benchmarks provided only vague measures. More nuanced thinking around implementation goals and the relative trade-offs of focusing on poverty versus other programmatic priorities is required. For example, the more practical questions for implementation should focus on relative priorities between urban and rural settings, static or adaptive locations for service provision, the need to address humanitarian settings (refugee camps, etc.), relative regional priorities, and trade-offs with other equity dimensions. Other relevant dimensions of disadvantage, such as caste structures, LGBTQ + , sex workers and other marginalised groups and how they intersect were not covered in the PbR mechanism. Both MSI and IPPF have long histories of identifying and serving vulnerable, disadvantaged and excluded service users. However, the experience of WISH shows how complex this can be and highlights the risks of a narrow and imperfect approach (particularly for poverty measurement). In addition, greater consideration of defining equity priorities at a system-level and in the sustainability work of the programme would be beneficial and ensure that other elements of equity are not lost.

This study had some limitations. First, the selection of only three countries for case studies may mean that the findings cannot be generalised across the WISH programme. In addition, the choice of case studies was purposeful and this raised the risk of self-selection bias regarding which countries to analyse. Likewise, the picking of respondents was not random. Also, the presence of COVID-19 created a counterfactual challenge: it can be difficult to assess whether the programmatic effects of PbR, the PbR KPI results themselves, or the way in which FCDO and partners have dealt with the programme and the risks faced are due to the challenges of COVID-19 or to the effects of the PbR mechanism itself. The study did work to carefully distinguish the effects of PbR during the pandemic, but inevitably some uncertainties remain. Finally, inquiring into the perspectives of those who used the services provided might offer further insights. For example, did adolescent service users feel that the WISH programme was better than other programmes which did not have the same incentives?

## Conclusion

The WISH programme was successful in achieving its desired coverage (CYPs and Additional Users), and the use of PbR KPIs clearly focussed the programming of the implementing partners and incentivised several strategies to reach adolescents and people living in poverty. However, lack of resources and a focus on clinical and service delivery approaches may have limited the incentives effects and created certain trade-offs between coverage and equity. In addition, the use of global standard indicators for measuring equity effects on sub-national level programmes proved to be an imprecise instrument to measure equity.

## Data Availability

The supporting data can be made available upon reasonable request.

## Abbreviations

AEA: American Evaluation Association
AU: Additional User
CEI: Client Exit Interview
CYP: Couple Years of Protection
DFID: Department for International Development
FCDO: Foreign, Commonwealth and Development Office
FP: Family Planning
IPPF: International Planned Parenthood Federation
KII: Key Informant Interview
KPI: Key Performance Indicators
LNOB: Leave No One Behind
MPI: Multidimensional Poverty Index
MSI: Marie Stories International
PBF: Performance Based Financing
PbR: Payment Based Results
PPI: Poverty Probability Index
PPP: Purchasing Power Parity
PWD: People Living with Disabilities
SHR: Sexual and Reproductive Health
SRHR: Sexual and Reproductive Health and Rights
WISH: Women’s Integrated Sexual Health

## Footnotes

1 Originally WISH was set up by the UK’s Department for International Development (DFID), which was merged with the Foreign and Commonwealth Office (FCO) in September 2020, to become the FCDO.

2 Lot 1: MSI Reproductive Choices (prime); IPPF, DKT International, Ipas, Thinkplace, Options, and Leonard Chesire Disability. Lot 2/ WISH2Action: IPPF (prime), International Rescue Committee, Development Media International (DMI), Ipas, Humanity and Inclusion.

3 ‘DFID’ is used throughout this study where it is appropriate. The DFID-FCO merger to form the FCDO took place in September 2020, so references after that point are to ‘FCDO’, and before that point ‘DFID’ is used. So, at the time of the design and tendering of WISH (2017–2018), DFID is used.

4 As per the UK’s International Development Act 2002.

5 PbR would be applicable for fees but not operating expenses. Interested suppliers had to specify what proportion of fees they would place ‘at risk’, the maximum being 100%.

6 There was a difference between the treatment of the Adolescents (b) 15% PbR KPI between the two lots – for Lot 1, it was a weighted average across the portfolio (i.e., the proportion of total clients aged under 20); for Lot 2, it was a simple average across (non-FCAS) countries (i.e., the average reach of clients aged under 20 across the 12 non-FCAS countries).

7 At the time of programming this was the proportion of the population living under the USD 1.90 PPP benchmark, which was used across countries. Although this poverty line has been recently revised to USD 2.15 PPP by the World Bank. The World Development Indicator (WDI) being SI.POV.DDAY.

8 For example, if the USD 1.90 benchmark estimate had 60% in poverty, at least 60% as a proportion of service users should be under this benchmark. Performance below target was scaled linearly in the financial value of PbR applied.

9 National average poverty rates include the economically inactive population (generally children and the elderly). The average poverty rates of those who are economically active will therefore be lower than those of the overall population.
